# Pharmacological inhibition of receptor protein tyrosine phosphatase β/ζ decreases Aβ plaques and neuroinflammation in the hippocampus of APP/PS1 mice

**DOI:** 10.3389/fphar.2024.1506049

**Published:** 2024-12-06

**Authors:** Teresa Fontán-Baselga, Héctor Cañeque-Rufo, Elisa Rivera-Illades, Esther Gramage, José María Zapico, Beatriz de Pascual-Teresa, María Del Pilar Ramos-Álvarez, Gonzalo Herradón, Marta Vicente-Rodríguez

**Affiliations:** ^1^ Department of Health and Pharmaceutical Sciences, Faculty of Pharmacy, Universidad San Pablo-CEU, CEU Universities, Urbanización Montepríncipe, Madrid, Spain; ^2^ Department of Chemistry and Biochemistry, Faculty of Pharmacy, Universidad San Pablo-CEU, CEU Universities, Urbanización Montepríncipe, Madrid, Spain

**Keywords:** Alzheimer’s disease, RPTP β/ζ, MY10, pleiotrophin, neuroinflammation, neurodegeneration

## Abstract

Alzheimer’s disease (AD) is a major neurodegenerative disorder that courses with chronic neuroinflammation. Pleiotrophin (PTN) is an endogenous inhibitor of Receptor Protein Tyrosine Phosphatase (RPTP) β/ζ which is upregulated in different neuroinflammatory disorders of diverse origin, including AD. To investigate the role of RPTPβ/ζ in neuroinflammation and neurodegeneration, we used eight-to ten-month-old APP/PS1 AD mouse model. They were administered intragastrically with MY10, an inhibitor of RPTPβ/ζ, at different doses (60 and 90 mg/kg) every day for 14 days. Treatment with 90 mg/kg MY10 significantly reduced the number and size of amyloid beta (Aβ) plaques in the dorsal subiculum of the hippocampus of APP/PS1 mice. In addition, we observed a significant decrease in the number and size of astrocytes in both sexes and in the number of microglial cells in a sex-dependent manner. This suggests that RPTPβ/ζ plays an important role in modulating Aβ plaque formation and influences glial responses, which may contribute to improved Aβ clearance. In addition, MY10 treatment decreased the interaction of glial cells with Aβ plaques in the hippocampus of APP/PS1 mice. Furthermore, the analysis of proinflammatory markers in the hippocampus revealed that MY10 treatment decreased the mRNA levels of *Tnfa* and *Hmgb1*. Notably, treatment with MY10 increased *Bace1* mRNA expression, which could be involved in enhancing Aβ degradation, and it decreased *Mmp9* levels, which might reflect changes in the neuroinflammatory environment and impact Aβ plaque dynamics. These results support the therapeutic potential of inhibition of RPTPβ/ζ in modulating Aβ pathology and neuroinflammation in AD.

## 1 Introduction

Alzheimer’s disease (AD) is one of the most prevalent neurodegenerative diseases worldwide. It is currently estimated to affect more than 44 million people, which is expected to double by 2050 (Dumurgier and Sabia, 2020). This condition is characterized by the presence of two histopathological hallmarks in the central nervous system (CNS), senile plaques, protein aggregates of amyloid beta (Aβ) peptide and neurofibrillary tangles (NFT), aggregates of hyperphosphorylated tau protein, leading to cognitive impairment and dementia (Zhao and Huai, 2023). In the last years, these two features of the disease have been the main therapeutic targets for AD drug development. However, there is still no cure for the disease, so further studies of the different mechanisms involved in the disease are needed.

The dorsal subiculum is one of the earliest regions affected in AD (Ye et al., 2024). It is crucial for learning and memory processes in rodents, as it serves as the main pathway for information leaving the CA1 region of hippocampus (O'Mara and Aggleton, 2019; Frost et al., 2021) and is a key structure within the hippocampal formation, playing an important role in spatial representation and navigation information processing (de Melo et al., 2023). This early involvement of the dorsal subiculum in AD suggests that it may be particularly vulnerable to the harmful effects of chronic neuroinflammation, which plays a significant role in disease progression.

Chronic neuroinflammation is a common feature of numerous neurodegenerative diseases, such as AD, Parkinson´s disease (PD) or multiple sclerosis (Glass et al., 2010). However, despite existing evidence of the importance of neuroinflammation, there is still a lot of controversy regarding its role and relevance in AD (Calsolaro and Edison, 2016; Heneka et al., 2015). The CNS immune response detects the abnormal protein aggregation as harmful, leading to astrogliosis around senile plaques and morphological microglial changes leading to the secretion of pro-inflammatory cytokines (Boche and Nicoll, 2008). Normally, once the damage has ceased, glial cells would stop the inflammatory response and return to the basal state. Nevertheless, in the event of persistent damage, glial cells are activated long-term, leading to uncontrolled neuroinflammation that can lead to neuronal dysfunction and cell death, promoting the progression of the disease (Carriba and Comella, 2015). All in all, compounds that can modulate neuroinflammation and neuroimmune responses in the CNS, could be a potential therapeutic target for AD (Yu et al., 2021).

Pleiotrophin (PTN) is an important cytokine for CNS repair, neuronal survival, and differentiation (Herradón and Pérez-García, 2014). It is widely expressed during development, while its pattern of expression in adults is restricted to a few cell types in different organs, including the brain, where it is mainly expressed in neurons in healthy murine models (Deuel et al., 2002; Herradon et al., 2019; Silos-Santiago et al., 1996; Vanderwinden et al., 1992) being its highest expression in the CNS during embryonic and neonatal periods (Wang, 2020). However, after an injury or noxious stimulus, its expression increases in different cells including microglia and macrophages (Martin et al., 2011; Jin et al., 2009; Muramatsu, 2011; González-Castillo et al., 2014). PTN is overexpressed in different brain areas in situations with inflammatory component, as in brain damage due to ischemia, in neuropathic pain, after administration of different drugs of abuse such as amphetamine, alcohol and opioids, even in neurodegenerative processes, in senile plaques in the brain of patients with AD and in the substantia nigra of patients with PD (Herradón and Pérez-García, 2014; Alguacil and Herradón, 2015), suggesting a modulatory role of PTN in these processes. PTN is a potent modulator of neuroinflammation in different contexts (Herradon et al., 2019; Fernández-Calle et al., 2017; Vicente-Rodríguez et al., 2016; Rodríguez-Zapata et al., 2024; Rodríguez-Zapata et al., 2023) and binds to different receptors in many organs, being Receptor Protein Tyrosine Phosphatase β/ζ (RPTPβ/ζ) mainly expressed in the adult CNS in neurons and glial cells (Canoll et al., 1996; Shintani et al., 1998; Lafont et al., 2009). RPTPβ/ζ is important for neuronal and microglial viability (Del Campo et al., 2021), and it is the most relevant in modulating neuroinflammation (Herradon et al., 2019). Pleiotrophin binds to the extracellular domain of RPTPβ/ζ (Maeda et al., 1996; Maeda et al., 1999), inactivating its phosphatase activity and therefore increasing the phosphorylation levels of its substrates, such as TrkA (Shintani and Noda, 2008) and Fyn kinase (Pariser et al., 2005; Panicker et al., 2015), both with known roles in neuroinflammation.

To further characterize the functions of RPTPβ/ζ, we designed and synthesized MY10, a selective inhibitor of RPTPβ/ζ permeable to the blood-brain barrier (BBB). MY10 interacts with the intracellular domain PD1 of RPTPβ/ζ, and it inactivates its tyrosine phosphatase activity, simulating the inhibitory action of PTN on this receptor (Pastor et al., 2018).

Based on these considerations, we aim to demonstrate that RPTPβ/ζ modulates neuroinflammation and neurodegeneration, using the selective inhibitor of RPTPβ/ζ, MY10, in a mouse model of AD.

## 2 Materials and methods

### 2.1 Animals

Heterozygous APPswe/PS1De9 (APP/PS1) double-transgenic female and male mice with a C57BL/6 background were used in the present study. The mice were divided randomly and housed in a specific pathogen-free room at 22°C ± 1°C with 12 h light/dark cycles, with free access to water and food. All the animals were handled and maintained in accordance with the European Union Laboratory Animal Care Rules (2010/63/EU directive) and protocols were approved by the Animal Research Committee of CEU San Pablo University and by Comunidad de Madrid (PROEX 140.3/22).

### 2.2 Treatment

The selective inhibitor of RPTPβ/ζ (MY10) was synthesized as previously described (Pastor et al., 2018). Eight-to ten-months-old APP/PS1 mice were administered with MY10 (at doses of 60 or 90 mg/kg) or its vehicle (VEH; 10% dehydrated ethanol, 20% polysorbate 80, 70% PEG- 300) as a control. The treatment was carried out daily for 14 days by oral gavage.

### 2.3 Tissue collection

After 14 days of treatment, animals were sacrificed on day 15 (n = 4–9/group) by decapitation under CO2 exposure. The brains were removed and divided in two hemispheres. The right hemispheres were post-fixed in paraformaldehyde (PFA) 4% for 48 h and then incubated in sucrose 30% for immunofluorescence. The left hemispheres were freshly collected and conserved at −80°C.

### 2.4 Immunofluorescence

Fixed brain hemispheres from vehicle- and MY10-treated APP/PS1 mice (60 mg/kg and 90 mg/kg) (n = 4–9 group) were coronally cut at 30 μm thickness using a sliding microtome (Leica SM2010 R). Immunohistochemistry studies were performed on the dorsal subiculum of the hippocampus of each animal (Bregma −2.69 mm to −3.87 mm).

Triple immunofluorescence of Aβ (Aβ; Abcam, Cambridge, UK; Ab201060; 1:1,000), PTN (PTN; Santa Cruz Biotechnology, Texas, United States; sc-74443; 1:50) together with Iba1 a marker for microglia and macrophages (Iba1; Abcam, Cambridge, United Kingdom; Ab5076; 1:1,000); or GFAP for astrocytes (GFAP; Thermo Fisher Scientific, Massachusetts, United States; PA110004; 1:1,000), or NeuN for neurons (NeuN; Synaptic Systems, Gottingen, Germany; SYSY266006; 1:200) were performed to determine brain Aβ formation, PTN expression and glial responses.

Free floating sections were washed three times with PBS and three times with PBS-2% Triton X-100. After washes, sections were blocked with 5% Bovine serum albumin (BSA) in PBS-Triton X-100 for 40 min. After rinses with PBS, sections were incubated overnight at 4°C with a mix of primary antibodies (see Supplementary Table S1). After washes with PBS, sections were incubated for 2 h with the appropriate Alexa-conjugated secondary antibodies (see Supplementary Table S1). Subsequently, the sections were counterstained with 4′,6-diamidino-2-phenylindole (DAPI) and mounted with Fluoromont^®^ mounting medium (Thermo Fisher Scientific; Massachusetts, United States; 00–4958–02).

Imaging was performed using a Leica DMI8 fluorescence confocal microscope. For relative quantification of immunofluorescence, one 380 μm × 380 µm photomicrograph containing series of ∼0.4 µm deep Z stacks, corresponding to ∼12 optical sections at 63X fields from the three fluorescence channels were captured from a dorsal subiculum area of the hippocampus (Bregma −2.69 mm to −3.87 mm) per animal (refraction index, 1.518). Construct composite images from each optical series by combining the images recorded through the different channels were obtained (image resolution: 512 × 512 pixels). The images were captured using the LAS × Core software (Leica Microsystems, Wetzlar, Germany; offline version).

### 2.5 Image analysis

For each photomicrograph, the total number of (i) Aβ count and Aβ % Area, (ii) GFAP + cells and GFAP % Area (for astrocytes), (iii) Iba1+ cells (microglia/macrophages), (iv) NeuN + cells (neurons), (v) GFAP + cells surrounding Aβ plaques, (vi) Iba1+ cells surrounding Aβ plaques and (vii) NeuN + cells surrounding Aβ plaques (viii) PTN % Area, (ix) GFAP+/PTN + cells (astrocytic cells expressing PTN), (x) Iba1+/PTN + cells (microglial/macrophages cells expressing PTN), (xi) NeuN+/PTN + cells (neuronal cells expressing PTN) were counted using ImageJ/Fiji software with the “Analyze Particle” function and colocalization analysis was performed by manual counting, with DAPI-stained nuclei as counterstain on a single brain slice from the subiculum of each animal, except from the quantification of PTN % Area, in which three slices from the three different immunofluorescences were analysed. For the Aβ plaques analysis, the ImageJ/Fiji threshold “Triangle” was used, followed by a removal of outliers. Finally, the “Analyze particle” function was used to obtain Aβ count and Aβ % Area. Each point in the graph represents the measurement in one brain slice.

ImageJ/Fiji software (NIH, Bethesda, MD, United States, Version 1.50 f) was used for the analysis of the subiculum of every subject. Images were escalated and converted into 8-bit grayscale. A threshold was adjusted for each cell type to reduce background noise.

### 2.6 Quantitative real-time PCR

The remaining hemispheres from vehicle-and MY10-treated APP/PS1 mice (60 mg/kg and 90 mg/kg) (n = 4–9 group) were dissected with the Mouse Brain matrix (Agnthos, Sweeden, 69–2165-1) in order to obtain the hippocampus. RNA isolation, First-strand cDNA synthesis and quantitative real-time PCR (qPCR) analysis were performed as previously described (Cañeque-Rufo et al., 2023). Briefly, RNA from the hippocampus was isolated using the Total RNA Isolation Kit (Nzytech, Lisbon, Portugal). First-strand cDNA was synthesized using the first-strand cDNA Synthesis Kit (Nzytech), and 1 μg of RNA were reverse-transcribed to DNA. qPCR analysis was performed using the SYBR green method (Quantimix Easy kit, Biotools, Madrid, Spain) in a CFX Opus 96 Real-Time System (Bio-Rad, Hercules, CA, United States). The relative expression of each gene was normalized using *Rpl13* and *Hprt* as housekeeping genes, and the data were analyzed by the Livak method. The primer sequences used, experimental conditions and additional information are shown in Supplementary Table S2.

### 2.7 Statistical analysis

Statistical analyses were performed using Graph-Pad Prism program version 8 (San Diego, CA, United States). The Shapiro-Wilk test was used for the normalities of the sample distribution. Data were analysed using a two-way ANOVA with treatment and sex as variables. When relevant, to better dissect the effect of each variable, we used a one-way ANOVA, excluding the non-significant variable if the two-way ANOVA results allowed it. Significant differences were analyzed by a Bonferroni’s Post-hoc only when the interaction between the variables were significant in the case of two-way ANOVA and always in the case of one-way ANOVA. Data are presented as mean ± standard error of the mean (S.E.M.).

## 3 Results

### 3.1 Inhibition of RPTPβ/ζ reduces Aβ plaques formation and glial responses in APP/PS1 mice

**FIGURE 1:**
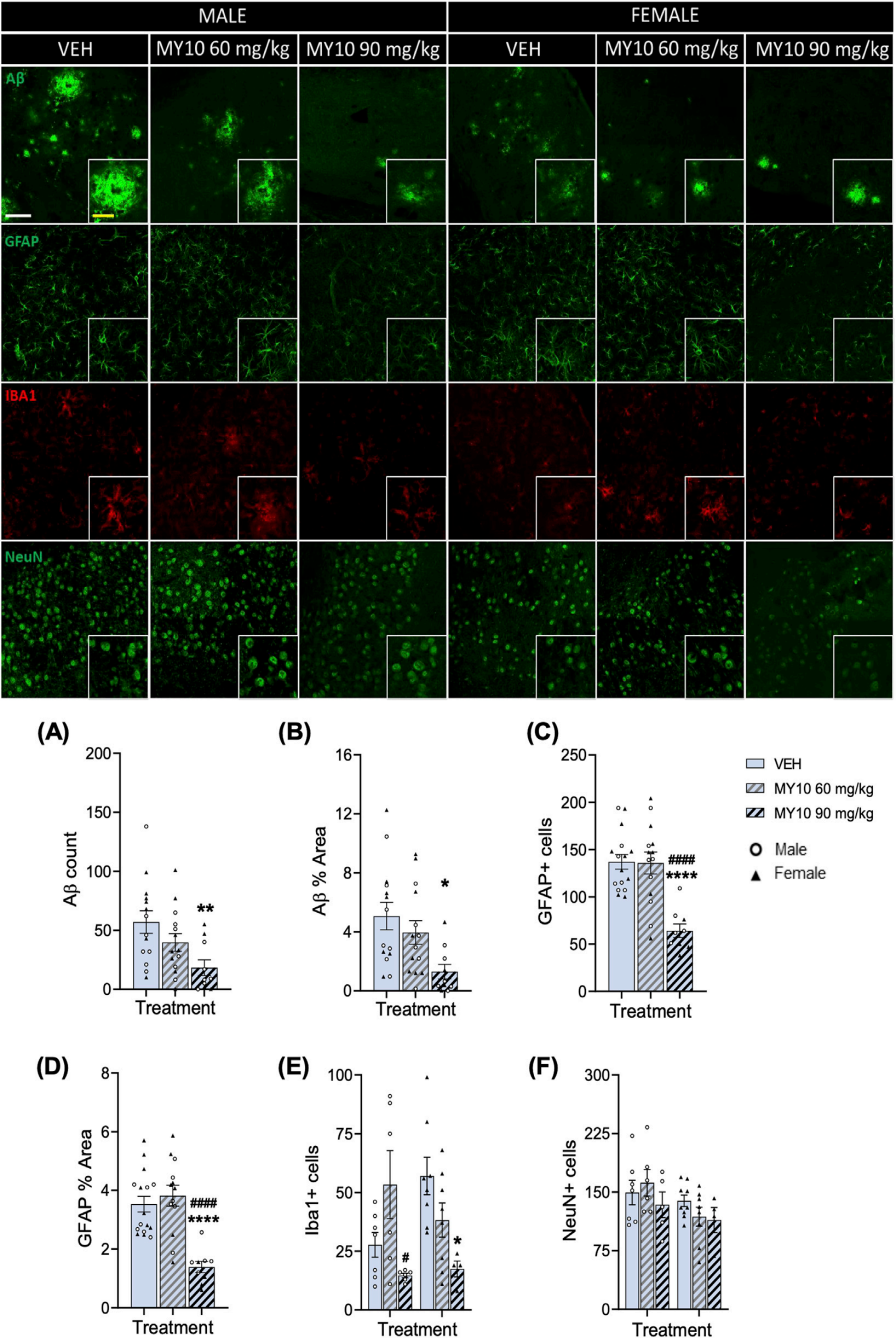
MY10 treatment effects on Aβ plaques, astrocytes (GFAP), microglia (Iba1) and neurons (NeuN). Representative confocal photomicrographs showing Aβ (green), GFAP (green), Iba1 (red) and NeuN (green) fluorescence from dorsal subiculum of the different experimental groups. Quantification of the number of Aβ plaques **(A)**, Aβ % Area **(B)**, number of GFAP + cells **(C)**, GFAP % Area **(D)**, number of Iba1+ cells **(E)**, number of NeuN + cells **(F)** per subiculum of APP/PS1 male and female mice treated with Vehicle (VEH), MY10 60 mg/kg or MY10 90 mg/kg. Data are presented as mean ± SEM (n = 4–9 APP/PS1 mice/treatment). Each point represents the measurement in one brain slice. *P<0.05; **p <0.01; ****p <0.0001 vs. VEH. #*p* <0.05; ####*p* <0.0001 vs. MY10 60 mg/kg. White scale bar 100 µm. Yellow scale bar 50 µm.

First, in immunohistochemistry studies, we analyzed the effects of treatment with MY10 on Aβ plaques and glial responses in male and female APP/PS1 mice (Figure 1). Two-way ANOVA revealed a significant effect of the treatment on Aβ count (F (2, 32) = 4.347; *p* = 0.0214) and Aβ % Area (F (2, 32) = 4.390; *p* = 0.0207). However, we did not observe a significant effect of sex or a significant interaction between variables. Thus, to better analyze the effect of treatment, we performed a one-way ANOVA excluding the sex variable. We detected significant differences in Aβ count (Figure 1A; F (2, 35) = 4.977; *p* = 0.0125) and Aβ % Area (Figure 1B; F (2, 35) = 4.995; *p* = 0.0124). Post hoc analysis revealed that treatment with 90 mg/kg MY10 significantly reduced Aβ compared to vehicle-treated APP/PS1 mice (Figures 1A, B). Two-way ANOVA revealed a significant effect of the treatment on the number of GFAP + cells (Figure 1C; F (2, 33) = 14.75; *p* <0.0001) and GFAP % Area (Figure 1D; F (2, 32) = 15.53; *p* <0.0001). Again, we did not observe a significant effect of sex or a significant interaction between variables. One-way ANOVA excluding sex variable revealed significant differences in the number of GFAP + cells (Figure 1C; F (2, 36) = 15.31; P<0.0001) and in the GFAP % Area (Figure 1D; F (2, 35) = 15.98; *p* <0.0001). Post hoc analysis revealed that treatment with 90 mg/kg MY10 significantly decreased the number of GFAP + cells and GFAP % Area compared to vehicle-treated and 60 mg/kg MY10-treated APP/PS1 mice. Regarding the number of Iba1+ cells, two-way ANOVA revealed a significant effect of the treatment (Figure 1 E; F (2, 33) = 6.652; *p* = 0.0037) and a significant interaction between sex and treatment (Figure 1 E; F (2, 33) = 4.171; *p* = 0.0243). Post hoc analysis showed that treatment with 90 mg/kg MY10 decreased the number of Iba1+ cells compared to 60 mg/kg MY10 in males and compared to the vehicle-treated female APP/PS1 mice. In contrast, in the two-way ANOVA of NeuN + cells, there was a significant effect in the sex (Figure 1 F; F (1,33) = 4.561; *p* = 0.0402) but no significant differences were observed in the treatment nor in the interaction.

### 3.2 Inhibition of RPTPβ/ζ reduces the interaction of glial cells with Aβ plaques and decreases PTN expression in APP/PS1 mice

**FIGURE 2:**
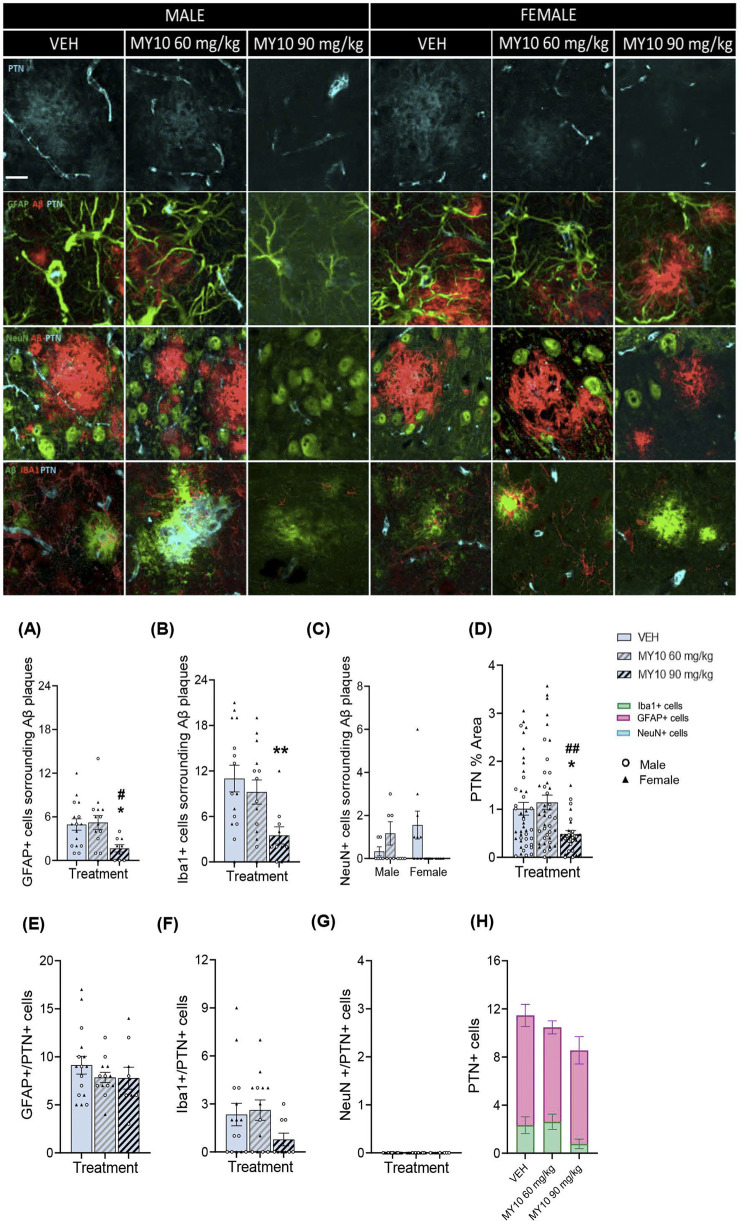
Effects of MY10 treatment on the colocalization of pleiotrophin (PTN) and Aβ plaques with astrocytes (GFAP), microglia (Iba1) and neurons (NeuN). Representative confocal photomicrographs showing Aβ, PTN, GFAP, Iba1 and NeuN fluorescence from the dorsal subiculum of the different experimental groups. Quantification of the number of GFAP + cells surrounding Aβ plaques **(A)**, number of Iba1+ cells surrounding Aβ plaques **(B)**, number of NeuN + cells surrounding Aβ plaques **(C)**, % area PTN + cells **(D)**, number of GFAP+/PTN + cells **(E)** number of Iba1+/PTN + cells **(F)**, number of NeuN+/PTN + cells **(G)**, number of PTN + cells in Iba1+, GFAP+ and NeuN + cells **(H)** per subiculum of APP/PS1 male and female mice treated with Vehicle (VEH), MY10 60 mg/kg or MY10 90 mg/kg. Data are presented as mean ± SEM (n = 4–9 APP/PS1 mice/treatment). Each point represents the measurement in one brain slice. *P<0.05; **p <0.01 vs. VEH. #P<0.05; ##*p* <0.01 vs. MY10 60 mg/kg. White scale bar 25 µm.

To study the effect of RPTPβ/ζ inhibition on the interaction of glial cells and neurons with Aβ plaques and on PTN expression in astrocytes, microglia and neurons, colocalization analyses were performed (Figure 2). Two-way ANOVA revealed a significant effect of the treatment on the number of GFAP + cells surrounding Aβ plaques (Figure 2A; F (2, 33) = 4.020; *p* = 0.0274); however, we did not detect significant sex differences. One-way ANOVA excluding sex factor revealed a significant effect of the treatment on the number of GFAP + cells surrounding Aβ plaques (Figure 2A; F (2, 36) = 4.353; *p* = 0.0203). Treatment with 90 mg/kg MY10 significantly reduced the number of GFAP + cells surrounding Aβ plaques compared to vehicle- and 60 mg/kg MY10-treated APP/PS1 mice. Lastly, two-way ANOVA revealed a significant effect of the treatment on the number of Iba1+ cells surrounding Aβ plaques (Figure 2B; F (2, 33) = 4.447; *p* = 0.0195), but no significant differences were observed between sexes. One-way ANOVA excluding the sex variable revealed a significant effect of the treatment in Iba1+ cells surrounding Aβ plaques (Figure 2B; F (2, 36) = 5.171; *p* = 0.0106). Treatment with 90 mg/kg MY10 significantly reduced Iba1+ cells surrounding Aβ plaques compared to vehicle-treated mice. Finally, two-way ANOVA revealed significant differences in NeuN + cells surrounding Aβ plaques in the interaction between sex and treatment (Figure 2C; F (2,30 = 3.614; *p* = 0.0393), but there were no significant differences in the post-hoc analysis.

On the other hand, two-way ANOVA revealed a significant effect of the treatment with MY10 on PTN (Figure 2D; F (2, 31) = 4.884; *p* = 0.0143). However, we did not observe a significant effect of sex or a significant interaction between variables. Thus, to better analyze the effect of treatment, we performed a one-way ANOVA excluding the sex variable. We detected a significant effect of the treatment on PTN (Figure 2D; F (2, 109) = 5.459; *p* = 0.0055), showing a significant reduction in APP/PS1 mice treated with 90 mg/kg MY10 compared to vehicle- and 60 mg/kg MY10-treated APP/PS1 mice. We did not observe significant effects of the treatment or sex in GFAP+/PTN + cells (Figure 2E). However, treatment with 90 mg/kg MY10 tended to decrease the number of Iba1+/PTN + cells in APP/PS1 mice compared to those treated with vehicle (Figure 2F). In addition, we observed that PTN is not expressed in neurons in APP/PS1 mice, (Figure 2G). Interestingly, we observed that the number of GFAP + cells expressing PTN was higher than the number of Iba1+ cells expressing PTN in APP/PS1 mice (Figure 2H).

### 3.3 Effects of the inhibition of RPTPβ/ζ on the levels of neuroinflammatory markers in the hippocampus of APP/PS1 mice

**FIGURE 3:**
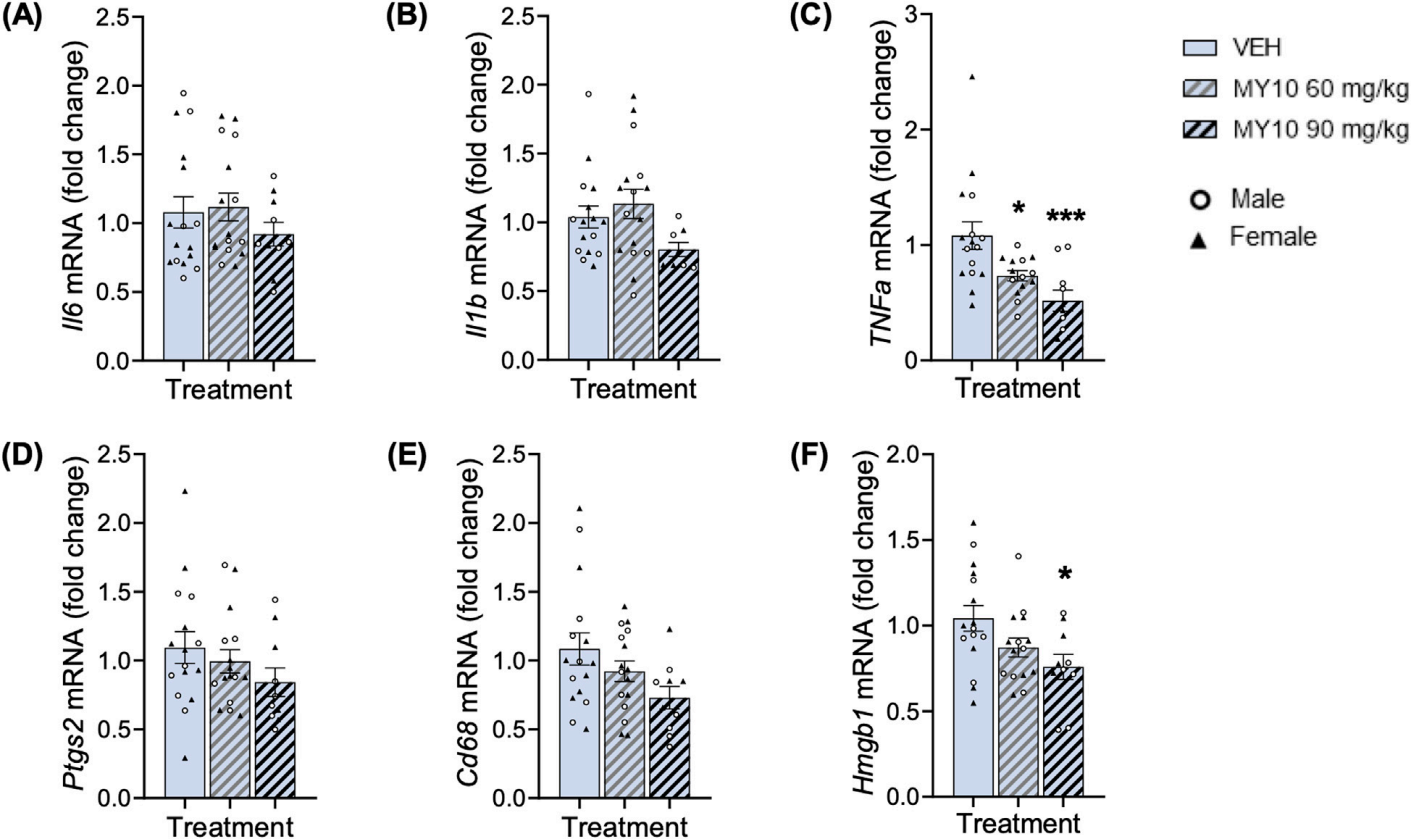
Effects of MY10 treatment on hippocampal neuroinflammatory markers. *Il6* (Interleukin 6) mRNA **(A)**, *Il1b* (Interleukin 1 Beta) mRNA **(B)**, *TNFa* (Tumor necrosis factor Alpha) mRNA **(C)**, *Ptgs2* (Prostaglandin-endoperoxide synthase 2) mRNA **(D)**, *Cd68* (Cluster of differentiation factor 68) mRNA **(E)**, *Hmgb1* (High mobility group-box 1) mRNA **(F)** levels in the hippocampus of APP/PS1 male and female mice treated with Vehicle (VEH), 60 mg/kg MY10 or 90 mg/kg MY10. Data are presented as mean ± SEM (n = 4–9 APP/PS1 mice/treatment). *P<0.05; ***p <0.001 vs. VEH.

We next analyzed the gene expression of neuroinflammatory markers in the hippocampus of mice across all experimental groups. Treatment with MY10 did not affect the mRNA levels of *Il6*, *Il1b*, *Ptgs2* or *Cd68* (Figures 3A, B, D, E). On the other hand, two-way ANOVA revealed a significant effect of the treatment on *Tnfa* mRNA levels (Figure 3C; F (2, 34) = 8.752; *p* = 0.0009), but we did not detect differences between sexes. The subsequent one-way ANOVA excluding sex variable confirmed the significant effect of treatment on the levels of *Tnfa* (Figure 3C; F (2, 37) = 8.555; *p* = 0.0009). We found a significant dose-dependent reduction of *Tnfa* mRNA levels in the hippocampi of APP/PS1 mice treated with MY10. In addition, two-way ANOVA did not reveal significant effects of the treatment or the sex on *Hmgb1* mRNA levels (Figure 3F). Thus, to better analyze the possible effect of treatment, we performed a one-way ANOVA excluding the sex variable. One-way ANOVA rendered a significant effect of the treatment (Figure 3F; F (2, 38) = 4.071; *p* = 0.0250), showing a significant reduction in the levels of *Hmgb1* mRNA in APP/PS1 mice treated with 90 mg/kg MY10 compared to vehicle-treated APP/PS1 mice. Taking all data together, we analyzed the pro-inflammatory gen signature (Supplementary Figure S1). One-way ANOVA revealed a significant effect of the treatment (Supplementary Figure S1B; F (2, 202) = 10.49; *p* <0.0001), showing a significant decrease of the pro-inflammatory mRNA signature in APP/PS1 mice treated with 90 mg/kg MY10, compared to those treated with vehicle and 60 mg/kg.

### 3.4 Inhibition of RPTPβ/ζ differentially regulates the expression of genes involved in the elimination of protein aggregates in the hippocampus

**FIGURE 4:**
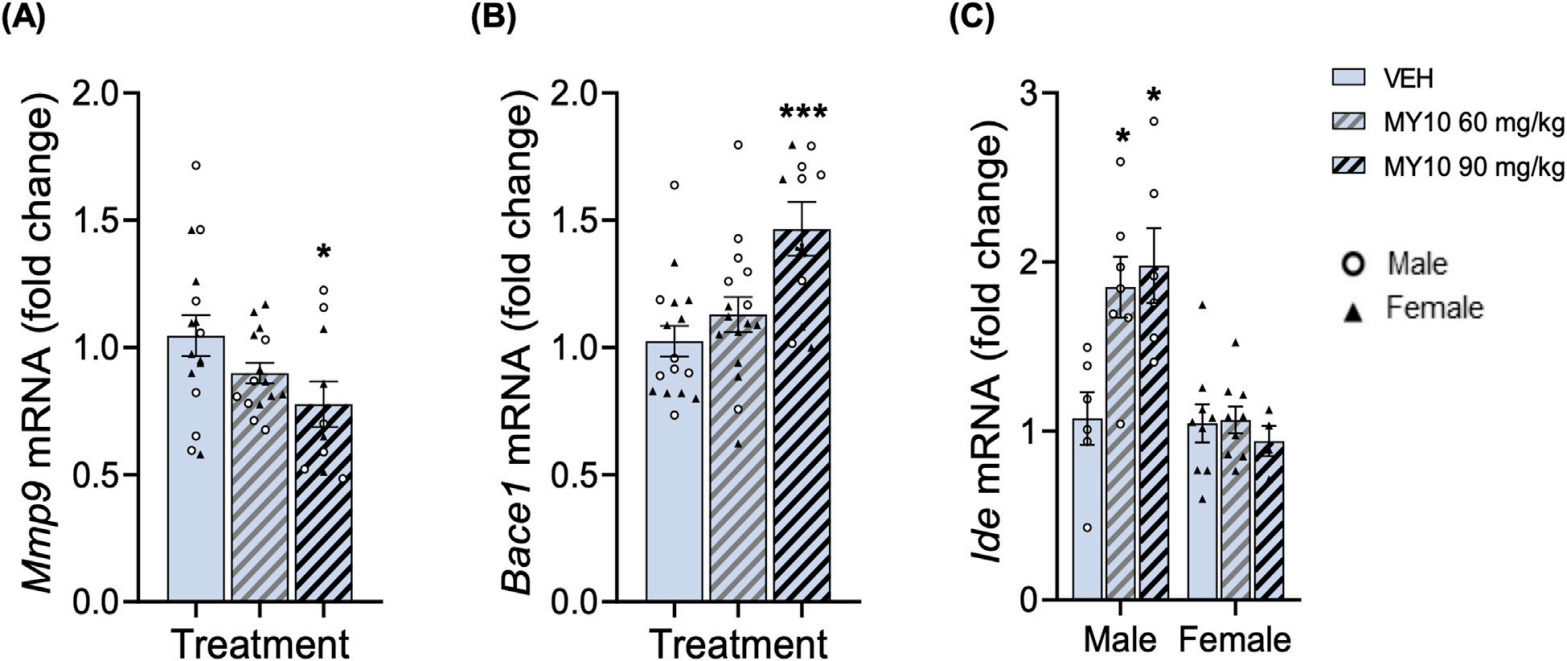
Effects of MY10 treatment on hippocampal gene expression involved in the elimination of protein aggregates. *Mmp9* (Metalloprotease 9) mRNA **(A)**. *Bace1* (Beta-secretase 1) mRNA **(B)**. *Ide* (Insulin-degrading enzyme) mRNA **(C)** in the hippocampus of APP/PS1 male and female mice treated with Vehicle (VEH), 60 mg/kg MY10 or 90 mg/kg MY10. Data are presented as mean ± SEM (n = 4-9 APP/PS1 mice/treatment). *p <0.05; ***p <0.001 vs. VEH.

The study of expression of genes involved in the elimination of protein aggregates showed relevant differences among experimental groups (Figure 4). Two-way ANOVA did not reveal significant effects of the treatment or the sex on *Mmp9* mRNA levels (Figure 4A). Thus, to better analyze the effect of treatment, we performed a one-way ANOVA excluding the sex variable that revealed a significant effect of the treatment (Figure 4A; F (2, 38) = 3.402; *p* = 0.0437). The data showed a significant decrease in *Mmp9* mRNA levels in APP/PS1 mice treated with 90 mg/kg MY10 compared to control mice. On the other hand, two-way ANOVA showed a significant effect of treatment with MY10 on the mRNA levels of Beta-secretase 1 (*Bace1*) (Figure 4B; F (2, 36) = 7.553; *p* = 0.0018); however, we did not detect a significant effect of sex. One-way ANOVA excluding sex factor showed a significant effect of treatment with MY10 on *Bace1* mRNA levels (Figure 4B; F (2, 39) = 8.045; *p* = 0.0012), showing a significant increase in the levels of *Bace1* in APP/PS1 mice treated with 90 mg/kg MY10 compared to vehicle-treated mice (Figure 4B). Lastly, two-way ANOVA revealed a significant effect of the treatment (Figure 4C; F (2, 35) = 5.108; *p* = 0.0113), a significant effect of sex (F (1, 35) = 25.32; *p* <0.0001) and a significant interaction between both variables (Figure 4C; F (2, 35) = 6.164; *p* = 0.0051) on the mRNA levels of *Ide*. Treatment with MY10 significantly increased the levels of expression of *Ide* only in the hippocampi of male APP/PS1 mice (Figure 4C).

## 4 Discussion

Alzheimer’s disease is a major public health concern worldwide. Every year, there are almost 10 million new cases, and there is still no cure (Passeri et al., 2022). There is an urgent need to find new therapeutic targets for this disease. Recent studies point out that modulating neuroinflammation seems to represent a valuable therapeutic approach. Therefore, compounds that modulate the immune response in the CNS may have therapeutic potential in AD. In this context, we previously demonstrated that the PTN/RPTPβ/ζ axis regulates glial responses and neuroinflammation induced by different stimuli (Herradon et al., 2019; Fernández-Calle et al., 2017; Vicente-Rodríguez et al., 2016). In the present work, we aimed to test the impact of RPTPβ/ζ inhibition with MY10 on neuroinflammation and neurodegeneration in AD, using the APP/PS1 animal model. Our findings reveal, for the first time, that treatment with MY10 significantly reduced the number and size of Aβ plaques in the dorsal subiculum of the hippocampus of APP/PS1 mice, suggesting that RPTPβ/ζ plays an important role modulating Aβ plaques formation in the hippocampus. The CNS immune response detects the abnormal protein aggregation as harmful, leading to astrogliosis and morphological microglial changes around senile plaques, resulting in pro-inflammatory cytokines secretion (Boche and Nicoll, 2008). Prolonged glial cells activation, causes uncontrolled neuroinflammation, neuronal dysfunction, and cell death, which promote disease progression (Carriba and Comella, 2015). On the other hand, the clearance of Aβ from the brain involves the active participation of glial cells amongst others (Yoon and Jo, 2012). They surround compacted Aβ plaques, forming a barrier to prevent the generation of new Aβ monomers and protect neurons (Ullah and Lee, 2023). Insufficient clearance of Aβ has been identified as the major pathological mechanism of AD (Cai et al., 2023). Given this context, our study aimed to test the possibility that RPTPβ/ζ inhibition with MY10 modulates glial responses in APP/PS1 mice. Our results indicate that treatment with MY10 decreased the number and size of astrocytes, as well as the number of microglial cells in male and female APP/PS1 mice. It is known that activated glial cells like microglia and astrocytes are key in promoting a neuroinflammatory response that can be the neuropathological event leading to neurodegeneration in AD (Fakhoury, 2018). Many reactive glial cells are found near senile plaques in AD patients, which suggests the role of these cells in the pathogenesis of the disease (Zotova et al., 2011). In this study, the decreased numbers of glial cells caused by MY10 treatment are associated with a reduction of Aβ, suggesting that a diminished persistent glial reactivity against this pathologic protein aggregation may contribute to mitigate the progression of neuroinflammation and neurodegeneration in the context of the disease. However, not only the reduction of gliosis is important but the proximity of glial cells to Aβ plaques and the type of molecules they express.

PTN is primarily expressed in the CNS but is significantly upregulated in various cells, including microglia and inflammatory macrophages, following injury (Martin et al., 2011; Jin et al., 2009; Muramatsu, 2011; González-Castillo et al., 2014). In APP/PS1 mice, we observed that PTN was predominantly expressed in astrocytes and, to a lesser extent, in microglia. Treatment with MY10 reduced the overall PTN marked area. Recent studies demonstrate that PTN is accumulated in senile plaques, and it has an impact on amyloid deposition by accelerating senile plaques aggregation (Levites et al., 2024). This suggests that MY10 treatment could reduce senile plaque aggregation by decreasing PTN expression. Specifically, MY10 tended to decrease PTN expression in microglial cells, but not in astrocytes, suggesting different regulatory mechanisms and cellular responses in astrocytes and microglia in AD. As expected, both astrocytes and microglia were spatially associated with Aβ plaques in APP/PS1 mice. Here, we show that MY10 treatment significantly reduced the number of astrocytes and microglial cells surrounding Aβ plaques in APP/PS1 mice. These results strongly support the modulation of neuroinflammation through RPTPβ/ζ inhibition and its potential association with the clearance of Aβ plaques in the hippocampus.

In response to Aβ plaque accumulation, microglia produces proinflammatory cytokines, leading to chronic neuroinflammation (Hansen et al., 2018). Interestingly, in the hippocampus of APP/PS1 mice, *Tnfa* and *Hmgb1* mRNA expression levels were significantly reduced by MY10. On the other hand, *Il6*, *Il1b*, *Ptgs2* and *Cd68* expression levels did not seem to be significantly modulated by MY10, suggesting that RPTPβ/ζ inhibition with MY10 modulates neuroinflammation by regulating *Tnfa* and *Hmgb1* mRNA expression levels in APP/PS1 mice. TNFα and HMGB1 are key pro-inflammatory molecules implicated in neuroinflammation, which plays a crucial role in AD progression. Several anti-inflammatory treatments targeting microglial activation have been shown to significantly decrease TNFα expression, reducing synaptic dysfunction and cognitive impairment in AD (Decourt et al., 2017). Similarly, extracellular HMGB1 is thought to contribute to AD pathology by inhibiting microglial phagocytosis and stabilizing Aβ42 oligomers. It has been demonstrated that inhibition of HMGB1 reduces neuroinflammation and enhances Aβ clearance (Fang et al., 2012). These findings support the idea that pharmacological treatments that modulate these inflammatory pathways, can suppress the activation of glial cells and promote Aβ clearance.

Microglia and astrocytes also promote the breakdown of Aβ fibrils and oligomers by secreting multiple Aβ-degrading enzymes (Abud et al., 2017) such as BACE1 (Ulku et al., 2023; Hampel et al., 2021), MMP9 (Hernandes-Alejandro et al., 2020) and IDE (Tian et al., 2023). Interestingly, we found that MY10 differentially modulates the hippocampal mRNA expression levels of these enzymes. Treatment with MY10 significantly increased *Bace1* mRNA expression in the hippocampus compared to vehicle-treated APP/PS1 mice. The beta-site amyloid precursor protein (APP) cleaving enzyme BACE1 has been known for years for its amyloidogenic activity, contributing to the production of Aβ peptides (Hampel et al., 2021). However, recent studies have established an amyloidolytic activity of BACE1, degrading longer Aβ peptides into a non-toxic Aβ34 intermediate (Ulku et al., 2023). This dual functionality of BACE1 suggests a complex regulatory role in amyloid metabolism. Specifically, while an excess of APP promotes the amyloidogenic Aβ peptide production, an excess of BACE1 facilitates increased Aβ peptides degradation (Liebsch et al., 2019). In this context, MY10 treatment may modulate Aβ plaques formation by enhancing the amyloidolytic activity of BACE1, thereby promoting the degradation of potentially toxic Aβ peptides.

Recent studies have elucidated that MMP9 contributes to the clearance of Aβ by degrading amyloid plaques and facilitating their removal from the brain (Fragkouli et al., 2014). However, our findings demonstrate that treatment with MY10 reduces the levels of *Mmp9* in the hippocampus of APP/PS1 mice. MMP9 is also involved in various physiological and pathological processes beyond Aβ degradation, such as inflammation. A decrease in *Mmp9* could also reflect changes in the neuroinflammatory environment or alterations in tissue homeostasis, which might indirectly affect Aβ plaque dynamics (Fujimoto et al., 2008). Inhibition of MMP9 facilitates Aβ clearance across the BBB and it also decreases tissue damage, neutrophil infiltration, oxidative stress and neuronal degeneration (Ringland et al., 2021; Wang and Tsirka, 2005; Romanic et al., 1998; Rosenberg et al., 1998). MMP9 binds and proteolyzes lipoprotein receptors inducing ectodomain shedding and reducing the ability to transport Aβ out of the brain (Shackleton et al., 2019). Further studies are needed to unravel if and how MY10-induced reduction of *Mmp9* levels is involved in the beneficial effects of MY10 treatment in the APP/PS1 mouse model.

In addition, hippocampal *Ide* mRNA expression was upregulated in male mice treated with MY10, whereas no significant differences were observed in females. IDE cleaves several peptides, such as insulin and Aβ (Qiu et al., 1998). However, its relevance lies in the fact that it is the primary soluble Aβ degrading enzyme at neutral pH in the human brain (Baranello et al., 2015; Dorfman et al., 2010). Accordingly, reduced IDE activity has been linked to increased Aβ accumulation and AD pathology (Tian et al., 2023). It is known that sex hormones modulate Aβ via induction of IDE among others (George et al., 2013). Nevertheless, the gender-specific increase in *Ide* activity in MY10-treated male mice needs further investigation and underscores the importance of considering biological sex in AD research and treatment development.

While this study provides important insights, certain limitations should be acknowledged. The temporal scope of our observations does not clarify whether the reduction in Aβ deposition reflects a lasting effect or a delay in progression, highlighting the need for further longitudinal studies. In addition, the precise mechanisms underlying these effects of MY10 remain unclear. Our findings are consistent with previous work in different cell lines and *in vivo* models; However, we did not directly examine the effects of MY10 on RPTPβ/ζ activity in primary neural cell types, which could render a deeper understanding of the mechanisms underlying these effects. Future studies are needed to address these limitations and build upon the current findings.

This study provides for the first time convincing evidence that RPTPβ/ζ inhibition with MY10 significantly reduces Aβ plaque formation, which seems related to the capacity of MY10 to regulate glial responses and the proinflammatory signal characteristic of AD, and to modulate the expression of Aβ aggregate-degrading enzymes. The data support that the PTN/RPTPβ/ζ signalling pathway could be a novel therapeutic target in AD.

## Data Availability

The original contributions presented in the study are included in the article/Supplementary Material, further inquiries can be directed to the corresponding authors.

## Abbreviations

AD: Alzheimer’s disease; APP/PS1: Heterozygous APPswe/PS1De9 double-transgenic mice; APP: Amyloid precursor protein; Aβ: Amyloid beta; *Bace1*: Beta-secretase 1; BBB: Blood-brain barrier; BSA: Bovine serum albumin; *Cd68*: Cluster of differentiation factor 68; CNS: Central nervous system; DAPI: 4′,6-Diamidino-2-phenylindole; *Hmgb1*: High mobility group-box 1; *Ide*: Insulin-degrading enzyme; *Il1b*: Interleukin 1 Beta; *Il6*: Interleukin 6; *Mmp9*: Metalloprotease 9; NFT: Neurofibrillary tangles; PD: Parkinson´s disease; PFA: Paraformaldehyde; *Ptgs2*: Prostaglandin-endoperoxide synthase 2; PTN: Pleiotrophin; qPCR: Quantitative real-time PCR; RPTP β/ζ: Receptor Protein Tyrosine Phosphatase β/ζ; SEM: Standard error of the mean; *TNFa*: Tumor necrosis factor Alpha; VEH: Vehicle.

## References

[B1] Abud E. M. Ramirez R. N. Martinez E. S. Healy L. M. Nguyen C. H. H. Newman S. A. (2017). iPSC-Derived human microglia-like cells to study neurological diseases. Neuron 94 (2), 278–293.e9. 10.1016/j.neuron.2017.03.042 28426964 PMC5482419

[B2] Alguacil L. F. Herradón G. (2015). Midkine and pleiotrophin in the treatment of neurodegenerative diseases and drug addiction. Recent Pat. CNS Drug Discov. 10 (1), 28–33. 10.2174/1574889810666150326103916 25808239

[B3] Baranello R. J. Bharani K. L. Padmaraju V. Chopra N. Lahiri D. K. Greig N. H. (2015). Amyloid-beta protein clearance and degradation (ABCD) pathways and their role in Alzheimer's disease. Curr. Alzheimer Res. 12 (1), 32–46. 10.2174/1567205012666141218140953 25523424 PMC4820400

[B4] Boche D. Nicoll J. A. (2008). The role of the immune system in clearance of Abeta from the brain. Brain Pathol. 18 (2), 267–278. 10.1111/j.1750-3639.2008.00134.x 18363937 PMC8095633

[B5] Cai W. Wu T. Chen N. (2023). The amyloid-beta clearance: from molecular targets to glial and neural cells. Biomolecules 13 (2), 313. 10.3390/biom13020313 36830682 PMC9953441

[B6] Calsolaro V. Edison P. (2016). Neuroinflammation in Alzheimer's disease: current evidence and future directions. Alzheimers Dement. 12 (6), 719–732. 10.1016/j.jalz.2016.02.010 27179961

[B7] Cañeque-Rufo H. Sánchez-Alonso M. G. Zuccaro A. Sevillano J. Ramos-Álvarez M. D. P. Herradón G. (2023). Pleiotrophin deficiency protects against high-fat diet-induced neuroinflammation: implications for brain mitochondrial dysfunction and aberrant protein aggregation. Food Chem. Toxicol. 172, 113578. 10.1016/j.fct.2022.113578 36566969

[B8] Canoll P. D. Petanceska S. Schlessinger J. Musacchio J. M. (1996). Three forms of RPTP-beta are differentially expressed during gliogenesis in the developing rat brain and during glial cell differentiation in culture. J. Neurosci. Res. 44 (3), 199–215. 10.1002/(SICI)1097-4547(19960501)44:3<199::AID-JNR1>3.0.CO;2-B 8723759

[B9] Carriba P. Comella J. X. (2015). Neurodegeneration and neuroinflammation: two processes, one target. Neural Regen. Res. 10 (10), 1581–1583. 10.4103/1673-5374.165269 26692848 PMC4660744

[B10] Decourt B. Lahiri D. K. Sabbagh M. N. (2017). Targeting tumor necrosis factor Alpha for alzheimer's disease. Curr. Alzheimer Res. 14 (4), 412–425. 10.2174/1567205013666160930110551 27697064 PMC5328927

[B11] Del Campo M. Fernández-Calle R. Vicente-Rodríguez M. Martín Martínez S. Gramage E. Zapico J. M. (2021). Role of receptor protein tyrosine phosphatase β/ζ in neuron-microglia communication in a cellular model of Parkinson's disease. Int. J. Mol. Sci. 22 (13), 6646. 10.3390/ijms22136646 34206170 PMC8269034

[B12] de Melo M. B. Daldegan-Bueno D. Favaro V. M. Oliveira M. G. M. (2023). The subiculum role on learning and memory tasks using rats and mice: a scoping review. Neurosci. Biobehav Rev. 155, 105460. 10.1016/j.neubiorev.2023.105460 37939978

[B13] Deuel T. F. Zhang N. Yeh H. J. Silos-Santiago I. Wang Z. Y. (2002). Pleiotrophin: a cytokine with diverse functions and a novel signaling pathway. Arch. Biochem. Biophys. 397 (2), 162–171. 10.1006/abbi.2001.2705 11795867

[B14] Dorfman V. B. Pasquini L. Riudavets M. López-Costa J. J. Villegas A. Troncoso J. C. (2010). Differential cerebral deposition of IDE and NEP in sporadic and familial Alzheimer's disease. Neurobiol. Aging 31 (10), 1743–1757. 10.1016/j.neurobiolaging.2008.09.016 19019493 PMC3266723

[B15] Dumurgier J. Sabia S. (2020). Epidemiology of Alzheimer's disease: latest trends. Rev. Prat. 70 (2), 149–151. 32877124

[B16] Fakhoury M. (2018). Microglia and astrocytes in alzheimer's disease: implications for therapy. Curr. Neuropharmacol. 16 (5), 508–518. 10.2174/1570159X15666170720095240 28730967 PMC5997862

[B17] Fang P. Schachner M. Shen Y. Q. (2012). HMGB1 in development and diseases of the central nervous system. Mol. Neurobiol. 45 (3), 499–506. 10.1007/s12035-012-8264-y 22580958

[B18] Fernández-Calle R. Vicente-Rodríguez M. Gramage E. Pita J. Pérez-García C. Ferrer-Alcón M. (2017). Pleiotrophin regulates microglia-mediated neuroinflammation. J. Neuroinflammation 14 (1), 46. 10.1186/s12974-017-0823-8 28259175 PMC5336633

[B19] Fragkouli A. Tsilibary E. C. Tzinia A. K. (2014). Neuroprotective role of MMP-9 overexpression in the brain of Alzheimer's 5xFAD mice. Neurobiol. Dis. 70, 179–189. 10.1016/j.nbd.2014.06.021 25008761

[B20] Frost B. E. Martin S. K. Cafalchio M. Islam M. N. Aggleton J. P. O'Mara S. M. (2021). Anterior thalamic inputs are required for subiculum spatial coding, with associated consequences for hippocampal spatial memory. J. Neurosci. 41 (30), 6511–6525. 10.1523/JNEUROSCI.2868-20.2021 34131030 PMC8318085

[B21] Fujimoto M. Takagi Y. Aoki T. Hayase M. Marumo T. Gomi M. (2008). Tissue inhibitor of metalloproteinases protect blood-brain barrier disruption in focal cerebral ischemia. J. Cereb. Blood Flow. Metab. 28 (10), 1674–1685. 10.1038/jcbfm.2008.59 18560439

[B22] George S. Petit G. H. Gouras G. K. Brundin P. Olsson R. (2013). Nonsteroidal selective androgen receptor modulators and selective estrogen receptor β agonists moderate cognitive deficits and amyloid-β levels in a mouse model of Alzheimer's disease. ACS Chem. Neurosci. 4 (12), 1537–1548. 10.1021/cn400133s 24020966 PMC3867967

[B23] Glass C. K. Saijo K. Winner B. Marchetto M. C. Gage F. H. (2010). Mechanisms underlying inflammation in neurodegeneration. Cell 140 (6), 918–934. 10.1016/j.cell.2010.02.016 20303880 PMC2873093

[B24] González-Castillo C. Ortuño-Sahagún D. Guzmán-Brambila C. Pallàs M. Rojas-Mayorquín A. E. (2014). Pleiotrophin as a central nervous system neuromodulator, evidences from the hippocampus. Front. Cell Neurosci. 8, 443. 10.3389/fncel.2014.00443 25620911 PMC4287103

[B25] Hampel H. Vassar R. De Strooper B. Hardy J. Willem M. Singh N. (2021). The β-secretase BACE1 in alzheimer's disease. Biol. Psychiatry 89 (8), 745–756. 10.1016/j.biopsych.2020.02.001 32223911 PMC7533042

[B26] Hansen D. V. Hanson J. E. Sheng M. (2018). Microglia in Alzheimer's disease. J. Cell Biol. 217 (2), 459–472. 10.1083/jcb.201709069 29196460 PMC5800817

[B27] Heneka M. T. Carson M. J. El Khoury J. Landreth G. E. Brosseron F. Feinstein D. L. (2015). Neuroinflammation in Alzheimer's disease. Lancet Neurol. 14 (4), 388–405. 10.1016/S1474-4422(15)70016-5 25792098 PMC5909703

[B28] Hernandes-Alejandro M. Montaño S. Harrington C. R. Wischik C. M. Salas-Casas A. Cortes-Reynosa P. (2020). Analysis of the relationship between metalloprotease-9 and tau protein in alzheimer's disease. J. Alzheimers Dis. 76 (2), 553–569. 10.3233/JAD-200146 32538846

[B29] Herradón G. Pérez-García C. (2014). Targeting midkine and pleiotrophin signalling pathways in addiction and neurodegenerative disorders: recent progress and perspectives. Br. J. Pharmacol. 171 (4), 837–848. 10.1111/bph.12312 23889475 PMC3925022

[B30] Herradon G. Ramos-Alvarez M. P. Gramage E. (2019). Connecting metainflammation and neuroinflammation through the PTN-MK-rptpβ/ζ Axis: relevance in therapeutic development. Front. Pharmacol. 10, 377. 10.3389/fphar.2019.00377 31031625 PMC6474308

[B31] Jin L. Jianghai C. Juan L. Hao K. (2009). Pleiotrophin and peripheral nerve injury. Neurosurg. Rev. 32 (4), 387–393. 10.1007/s10143-009-0202-8 19424734

[B32] Lafont D. Adage T. Gréco B. Zaratin P. (2009). A novel role for receptor like protein tyrosine phosphatase zeta in modulation of sensorimotor responses to noxious stimuli: evidences from knockout mice studies. Behav. Brain Res. 201 (1), 29–40. 10.1016/j.bbr.2009.01.025 19428613

[B33] Levites Y. Dammer E. B. Ran Y. Tsering W. Duong D. Abreha M. (2024). Integrative proteomics identifies a conserved Aβ amyloid responsome, novel plaque proteins, and pathology modifiers in Alzheimer's disease. Cell Rep. Med. 5 (8), 101669. 10.1016/j.xcrm.2024.101669 39127040 PMC11384960

[B34] Liebsch F. Kulic L. Teunissen C. Shobo A. Ulku I. Engelschalt V. (2019). Aβ34 is a BACE1-derived degradation intermediate associated with amyloid clearance and Alzheimer's disease progression. Nat. Commun. 10 (1), 2240. 10.1038/s41467-019-10152-w 31110178 PMC6527709

[B35] Maeda N. Ichihara-Tanaka K. Kimura T. Kadomatsu K. Muramatsu T. Noda M. (1999). A receptor-like protein-tyrosine phosphatase PTPzeta/RPTPbeta binds a heparin-binding growth factor midkine. Involvement of arginine 78 of midkine in the high affinity binding to PTPzeta. J. Biol. Chem. 274 (18), 12474–12479. 10.1074/jbc.274.18.12474 10212223

[B36] Maeda N. Nishiwaki T. Shintani T. Hamanaka H. Noda M. (1996). 6B4 proteoglycan/phosphacan, an extracellular variant of receptor-like protein-tyrosine phosphatase zeta/RPTPbeta, binds pleiotrophin/heparin-binding growth-associated molecule (HB-GAM). J. Biol. Chem. 271 (35), 21446–21452. 10.1074/jbc.271.35.21446 8702927

[B37] Martin Y. B. Herradón G. Ezquerra L. (2011). Uncovering new pharmacological targets to treat neuropathic pain by understanding how the organism reacts to nerve injury. Curr. Pharm. Des. 17 (5), 434–448. 10.2174/138161211795164130 21375486

[B38] Muramatsu T. (2011). Midkine: a promising molecule for drug development to treat diseases of the central nervous system. Curr. Pharm. Des. 17 (5), 410–423. 10.2174/138161211795164167 21375488 PMC3267162

[B39] O'Mara S. M. Aggleton J. P. (2019). Space and memory (far) beyond the Hippocampus: many subcortical structures also support cognitive mapping and mnemonic processing. Front. Neural Circuits 13, 52. 10.3389/fncir.2019.00052 31447653 PMC6692652

[B40] Panicker N. Saminathan H. Jin H. Neal M. Harischandra D. S. Gordon R. (2015). Fyn kinase regulates microglial neuroinflammatory responses in cell culture and animal models of Parkinson's disease. J. Neurosci. 35 (27), 10058–10077. 10.1523/JNEUROSCI.0302-15.2015 26157004 PMC4495236

[B41] Pariser H. Ezquerra L. Herradon G. Perez-Pinera P. Deuel T. F. (2005). Fyn is a downstream target of the pleiotrophin/receptor protein tyrosine phosphatase beta/zeta-signaling pathway: regulation of tyrosine phosphorylation of Fyn by pleiotrophin. Biochem. Biophys. Res. Commun. 332 (3), 664–669. 10.1016/j.bbrc.2005.05.007 15925565

[B42] Passeri E. Elkhoury K. Morsink M. Broersen K. Linder M. Tamayol A. (2022). Alzheimer's disease: treatment strategies and their limitations. Int. J. Mol. Sci. 23 (22), 13954. 10.3390/ijms232213954 36430432 PMC9697769

[B43] Pastor M. Fernández-Calle R. Di Geronimo B. Vicente-Rodríguez M. Zapico J. M. Gramage E. (2018). Development of inhibitors of receptor protein tyrosine phosphatase β/ζ (PTPRZ1) as candidates for CNS disorders. Eur. J. Med. Chem. 144, 318–329. 10.1016/j.ejmech.2017.11.080 29275231 PMC5817915

[B44] Qiu W. Q. Walsh D. M. Ye Z. Vekrellis K. Zhang J. Podlisny M. B. (1998). Insulin-degrading enzyme regulates extracellular levels of amyloid beta-protein by degradation. J. Biol. Chem. 273 (49), 32730–32738. 10.1074/jbc.273.49.32730 9830016

[B45] Ringland C. Schweig J. E. Eisenbaum M. Paris D. Ait-Ghezala G. Mullan M. (2021). MMP9 modulation improves specific neurobehavioral deficits in a mouse model of Alzheimer's disease. BMC Neurosci. 22 (1), 39. 10.1186/s12868-021-00643-2 34034683 PMC8152085

[B46] Rodríguez-Zapata M. Galán-Llario M. Cañeque-Rufo H. Sevillano J. Sánchez-Alonso M. G. Zapico J. M. (2023). Implication of the PTN/rptpβ/ζ signaling pathway in acute ethanol neuroinflammation in both sexes: a comparative study with lps. Biomedicines 11 (5), 1318. 10.3390/biomedicines11051318 37238989 PMC10215719

[B47] Rodríguez-Zapata M. López-Rodríguez R. Ramos-Álvarez M. D. P. Herradón G. Pérez-García C. Gramage E. (2024). Pleiotrophin modulates acute and long-term LPS-induced neuroinflammatory responses and hippocampal neurogenesis. Toxicology 509, 153947. 10.1016/j.tox.2024.153947 39255863

[B48] Romanic A. M. White R. F. Arleth A. J. Ohlstein E. H. Barone F. C. (1998). Matrix metalloproteinase expression increases after cerebral focal ischemia in rats: inhibition of matrix metalloproteinase-9 reduces infarct size. Stroke 29 (5), 1020–1030. 10.1161/01.str.29.5.1020 9596253

[B49] Rosenberg G. A. Estrada E. Y. Dencoff J. E. (1998). Matrix metalloproteinases and TIMPs are associated with blood-brain barrier opening after reperfusion in rat brain. Stroke 29 (10), 2189–2195. 10.1161/01.str.29.10.2189 9756602

[B50] Shackleton B. Ringland C. Abdullah L. Mullan M. Crawford F. Bachmeier C. (2019). Influence of matrix metallopeptidase 9 on beta-amyloid elimination across the blood-brain barrier. Mol. Neurobiol. 56 (12), 8296–8305. 10.1007/s12035-019-01672-z 31209784 PMC6842100

[B51] Shintani T. Noda M. (2008). Functions of receptor-type protein tyrosine phosphatase in the formation of retinal projection. Seikagaku 80 (8), 733–742. 18807498

[B52] Shintani T. Watanabe E. Maeda N. Noda M. (1998). Neurons as well as astrocytes express proteoglycan-type protein tyrosine phosphatase zeta/RPTPbeta: analysis of mice in which the PTPzeta/RPTPbeta gene was replaced with the LacZ gene. Neurosci. Lett. 247 (2-3), 135–138. 10.1016/s0304-3940(98)00295-x 9655611

[B53] Silos-Santiago I. Yeh H. J. Gurrieri M. A. Guillerman R. P. Li Y. S. Wolf J. (1996). Localization of pleiotrophin and its mRNA in subpopulations of neurons and their corresponding axonal tracts suggests important roles in neural-glial interactions during development and in maturity. J. Neurobiol. 31 (3), 283–296. 10.1002/(SICI)1097-4695(199611)31:3<283::AID-NEU2>3.0.CO;2-6 8910787

[B54] Tian Y. Jing G. Zhang M. (2023). Insulin-degrading enzyme: roles and pathways in ameliorating cognitive impairment associated with Alzheimer's disease and diabetes. Ageing Res. Rev. 90, 101999. 10.1016/j.arr.2023.101999 37414154

[B55] Ulku I. Liebsch F. Akerman S. C. Schulz J. F. Kulic L. Hock C. (2023). Mechanisms of amyloid-β34 generation indicate a pivotal role for BACE1 in amyloid homeostasis. Sci. Rep. 13 (1), 2216. 10.1038/s41598-023-28846-z 36750595 PMC9905473

[B56] Ullah R. Lee E. J. (2023). Advances in amyloid-β clearance in the brain and periphery: implications for neurodegenerative diseases. Exp. Neurobiol. 32 (4), 216–246. 10.5607/en23014 37749925 PMC10569141

[B57] Vanderwinden J. M. Mailleux P. Schiffmann S. N. Vanderhaeghen J. J. (1992). Cellular distribution of the new growth factor pleiotrophin (HB-GAM) mRNA in developing and adult rat tissues. Anat. Embryol. Berl. 186 (4), 387–406. 10.1007/BF00185989 1416088

[B58] Vicente-Rodríguez M. Rojo Gonzalez L. Gramage E. Fernández-Calle R. Chen Y. Pérez-García C. (2016). Pleiotrophin overexpression regulates amphetamine-induced reward and striatal dopaminergic denervation without changing the expression of dopamine D1 and D2 receptors: implications for neuroinflammation. Eur. Neuropsychopharmacol. 26 (11), 1794–1805. 10.1016/j.euroneuro.2016.09.002 27642078

[B59] Wang J. Tsirka S. E. (2005). Neuroprotection by inhibition of matrix metalloproteinases in a mouse model of intracerebral haemorrhage. Brain 128 (7), 1622–1633. 10.1093/brain/awh489 15800021

[B60] Wang X. (2020). Pleiotrophin: activity and mechanism. Adv. Clin. Chem. 98, 51–89. 10.1016/bs.acc.2020.02.003 32564788 PMC7672882

[B61] Ye Q. Gast G. Wilfley E. G. Huynh H. Hays C. Holmes T. C. (2024). Monosynaptic rabies tracing reveals sex- and age-dependent dorsal subiculum connectivity alterations in an alzheimer's disease mouse model. J. Neurosci. 44 (16), e1796232024. 10.1523/JNEUROSCI.1796-23.2024 38503494 PMC11026364

[B62] Yoon S. S. Jo S. A. (2012). Mechanisms of amyloid-β peptide clearance: potential therapeutic targets for alzheimer's disease. Biomol. Ther. Seoul. 20 (3), 245–255. 10.4062/biomolther.2012.20.3.245 24130920 PMC3794520

[B63] Yu T. W. Lane H. Y. Lin C. H. (2021). Novel therapeutic approaches for alzheimer's disease: an updated review. Int. J. Mol. Sci. 22 (15), 8208. 10.3390/ijms22158208 34360973 PMC8348485

[B64] Zhao J. Huai J. (2023). Role of primary aging hallmarks in Alzheimer’s disease. Theranostics 13 (1), 197–230. 10.7150/thno.79535 36593969 PMC9800733

[B65] Zotova E. Holmes C. Johnston D. Neal J. W. Nicoll J. A. Boche D. (2011). Microglial alterations in human Alzheimer's disease following Aβ42 immunization. Neuropathol. Appl. Neurobiol. 37 (5), 513–524. 10.1111/j.1365-2990.2010.01156.x 21166690

