# Supplementary material for: Pharmacological inhibition of receptor protein tyrosine phosphatase β/ζ decreases Aβ plaques and neuroinflammation in the hippocampus of APP/PS1 mice
Source: Front Pharmacol. 2024 Dec 6;15:1506049. doi: 10.3389/fphar.2024.1506049 (PMC11658987; doi:10.3389/fphar.2024.1506049)
Supplement: Supplementary file 1 [file DataSheet1.docx]

Supplementary Material

# Supplementary Data

## Supplementary Figures


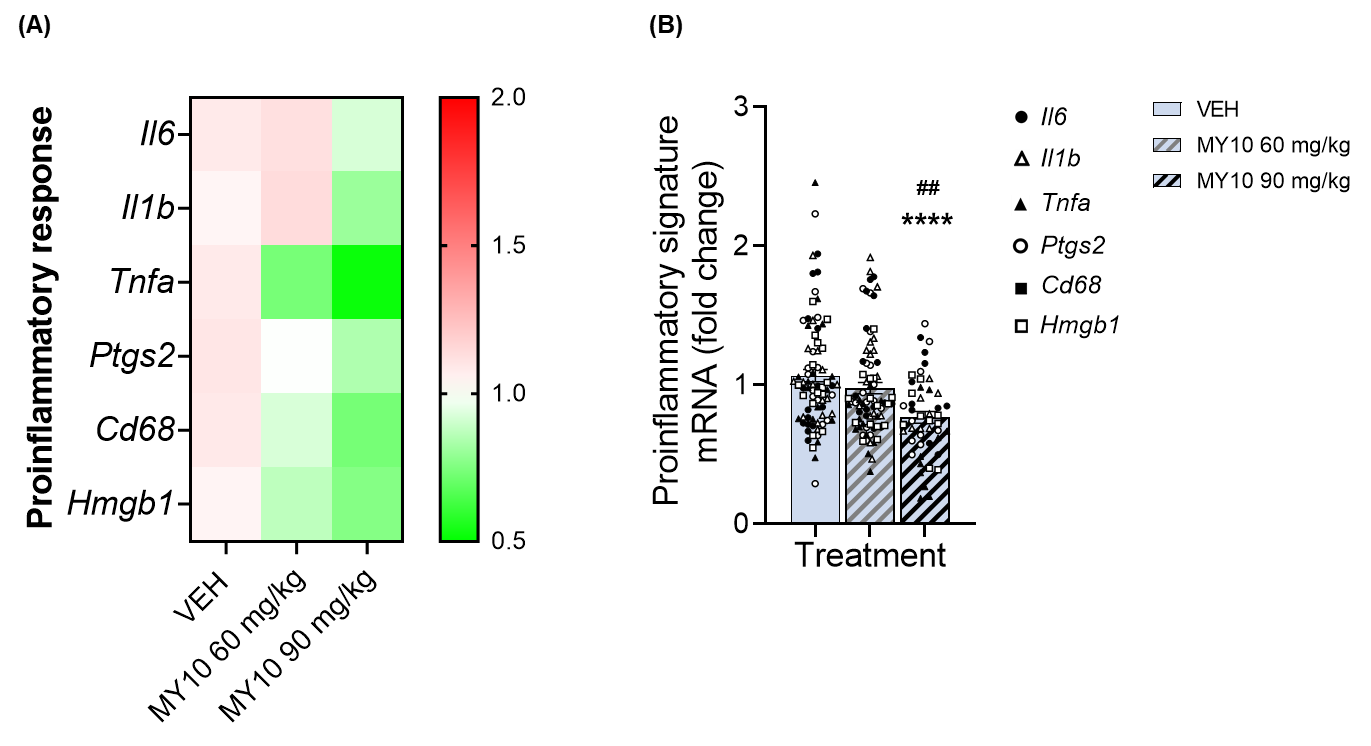


**Supplementary figure 1. Effects of MY10 treatment on hippocampal proinflammatory markers.** Heat map of proinflammatory response mRNA (A). Proinflammatory signature mRNA (B) in the hippocampus of APP/PS1 male and female mice treated with Vehicle (VEH), 60 mg/kg MY10 or 90 mg/kg MY10. Data are presented as mean ± SEM (n=4-9 APP/PS1 mice/treatment). ****P<0.0001 vs. VEH. ## p<0.01 vs. MY10 60 mg/kg.

## Supplementary Tables

**Supplementary table 1 Primary and secondary antibodies used for immunofluorescence.** NeuN (Neuronal nuclear antigen), PTN (pleiotrophin), Aβ (amyloid beta), GFAP (glial fibrillary acidic protein), Iba1 (Ionized calcium binding adaptor molecule 1).

| **Supplementary Table 1. Primary and secondary antibodies used for immunofluorescence** | | | |
| --- | --- | --- | --- |
| **Primary antibodies** | **Dilution** | **Supplier** | **Catalog number** |
| Chicken anti-NeuN | 1:200 | Synaptic Systems | #SYSY266006 |
| Mouse anti-PTN | 1:50 | Santa Cruz | #74443 |
| Rabbit anti- Aβ | 1:1000 | Abcam | #Ab201060 |
| Chicken anti-GFAP | 1:1000 | Thermo Fisher | #PA110004 |
| Goat anti-Iba1 | 1:1000 | Abcam | #Ab5076 |
| **Secondary antibodies** | **Dilution** | **Supplier** | **Catalog number** |
| Alexa Fluor 488 anti-chicken IgY | 1:600 | Jackson Immuno | #703545155 |
| Alexa Fluor 555 anti-mouse IgG | 1:1000 | Thermo Fisher | #A21422 |
| Alexa Fluor 647 anti-rabbit IgG | 1:600 | Jackson Immuno | #711605152 |
| Alexa Fluor 647 anti-goat IgG | 1:600 | Jackson Immuno | #705605147 |
| Alexa Fluor 555 anti-mouse IgG | 1:1000 | Abcam | #Ab150106 |
| Alexa Fluor 488 anti-rabbit IgG | 1:800 | Thermo Fisher | # A21206 |

**Supplementary table 2. Primer sets used for qPCR analysis.** Cd68: Cluster of differentiation factor 68; Ide: Insulin-degrading enzyme; Il1b: Interleukin 1 beta; Il6: Interleukin 6; Mmp9: Metalloprotease 9; Ptgs2: Prostaglandin-endoperoxide synthase 2; Rpl13: Ribosomal protein L13; Bace1 (Beta-secretase 1); Hprt1: Hypoxanthine phosphoribosyltransferase 1; Tnfa: Tumor necrosis factor alpha; Hmgb1: High mobility group box 1

| **Supplementary Table 2.** Primer sets used for qPCR analysis. | | | | | | |
| --- | --- | --- | --- | --- | --- | --- |
| **Gene** | **Primer Forward/Reverse** | **T_a_ (ºC)** | **Product lenght (bp)** | **%CG** | **Self complementarity** | **Self 3’**  **complementarity** |
| ***Cd68*** | 5'-TGGCGGTGGAATACAATGTG-3'  5'-GATGAATTCTGCGCCATGAA-3' | 61 | 67 | 50.00  45.00 | 5.00  6.00 | 3.00  4.00 |
| ***Ide*** | 5'-TGTTTCCACACACAGGCAAT-3'  5'-ACCTGTGAAAAGCCGAGAGA-3' | 60 | 156 | 45.00  50.00 | 4.00  2.00 | 2.00  0.00 |
| ***Il1b*** | 5'-GCTGAAAGCTCTCCACCTCA-3'  5'-AGGCCACAGGTATTTTGTCG-3' | 60 | 104 | 55.00  50.00 | 6.00  4.00 | 1.00  2.00 |
| ***Il6*** | 5'-TAGTCCTTCCTACCCCAATTTCC-3'  5'-TTGGTCCTTAGCCACTCCTTC-3' | 60 | 76 | 47.83  52.38 | 4.00  3.00 | 0.00  0.00 |
| ***Mmp9*** | 5'-GCATACTTGTACCGCTATGGT-3'  5'-TGTGATGTTATGATGGTCCC-3' | 60 | 225 | 47.62  45.00 | 6.00  3.00 | 2.00  2.00 |
| ***Ptgs2*** | 5'-ACGGAGAGAGTTCATCCCTGA-3'  5'-ACCCAGGTCCTCGCTTATGA-3' | 61 | 110 | 52.38  55.00 | 3.00  5.00 | 2.00  2.00 |
| ***Rpl13*** | 5'-GGTGCCCTACAGTTAGATACCAC-3'  5'-TTTGTTTCGCCTCCTTGGGTC-3' | 61 | 137 | 52.17  52.38 | 3.00  2.00 | 0.00  1.00 |
| ***Bace1*** | 5′-TCGCTGTCTCACAGTCATCC-3′  5′-AACAAACGGACCTTCCACTG-3′ | 60 | 161 | 55.00  50.00 | 5.00  3.00 | 1.00  3.00 |
| ***Hprt*** | 5’-TGCTCGAGATGTCATGAAGG-3’  5’-TATGTCCCCCGTTGACTGAT-3’ | 53 | 196 | 50.00  50.00 | 6.00  3.00 | 0.00  2.00 |
| ***Tnfa*** | 5'-AGGCACTCCCCCAAAAGATG-3'  5'-TGAGGGTCTGGGCCATAGAA-3' | 65 | 192 | 55.00  55.00 | 3.00  5.00 | 0.00  2.00 |
| ***Hmgb1*** | 5’-CGGAGAAACTTCAGACCGGA-3’  5’-CCCATGTTTAGTTGATTTTCCAGC-3’ | 60 | 178 | 55.00  41.67 | 4.00  4.00 | 2.00  2.00 |
